# Leptin, Leptin Receptor Concentrations and Free Leptin Index (FLI) in Polish Healthy Children and Adolescents

**DOI:** 10.34763/jmotherandchild.20252901.d-24-00049

**Published:** 2025-03-28

**Authors:** Joanna Gajewska, Grażyna Rowicka, Witold Klemarczyk, Ewa Głąb-Jabłońska, Jadwiga Ambroszkiewicz

**Affiliations:** Department of Screening Tests and Metabolic Diagnostics, Institute of Mother and Child, Warsaw 01-211, Kasprzaka 17a, Poland; Outpatient Gastroenterology Clinic, Institute of Mother and Child, Warsaw 01-211, Kasprzaka 17a Poland; Department of Nutrition, Institute of Mother and Child, Warsaw 01-211, Kasprzaka 17a Poland

**Keywords:** adipokines, leptin, soluble leptin receptor, healthy children

## Abstract

**Background:**

Leptin physiology in children is crucial for diagnosing and managing pediatric endocrine and metabolic disorders. The aim of this study was to assess the values of leptin, leptin receptor (sOB-R), and free leptin index (FLI) depending on age and sex in healthy Polish children and adolescents.

**Materials and methods:**

A total of 236 children and adolescents aged 1–18 years were recruited. Leptin and leptin receptor concentrations were determined by immunoenzymatic methods. FLI values were calculated as leptin divided by sOB-R concentrations. In 114 children between the ages of 5 and 10 years, a measurement of fat mass was assessed by dual-energy X-ray absorptiometry.

**Results:**

The studied groups of girls and boys were of similar age and did not differ in terms of weight, height, body mass index (BMI), BMI Z-score values, or leptin receptor concentrations. Leptin concentrations and FLI were higher by about 50% (p=0.006; p=0.051, respectively) in girls than boys. Positive correlations were found between leptin and age, BMI, and BMI Z-score values (r=0.562, r=0.563, p=0.397; p<0.001, respectively), and even stronger between FLI and age, BMI, and BMI Z-score values (r=0.670, r=0.632, p=0.409; p<0.001, respectively).

**Conclusions:**

The results concerning leptin and leptin receptor concentrations and FLI values in healthy individuals may be useful in clinical practice in early identification of children and adolescents with an unfavorable adipokine profile resulting in a predisposition to the development of obesity and obesity-related complications. These markers may also be helpful in monitoring therapy effectiveness in patients with obesity, diabetes, and metabolic syndrome.

## 1. Introduction

Leptin, a pivotal hormone, secreted mainly from white adipose tissue, has garnered significant attention since its identification in 1994 due to its fundamental role in regulating energy homeostasis, appetite, and metabolism. Structurally, leptin is a 16-kDa polypeptide typically consisting of a chain of 166 or 167 amino acids [1,2]. Its structure is analogous to that of long-chain helical cytokines, making leptin immunologically active. The biological importance of leptin extends beyond its central role in energy balance [3]. Its serum concentration was directly proportional to body mass index (BMI). Leptin acts as a key signal reflecting the body’s energy stores, thus influencing immune responses, reproductive function, and bone formation [4]. Dysregulation of leptin signaling has been implicated in a wide spectrum of metabolic and endocrine disorders, making it a crucial subject of study in both clinical and research contexts [5,6]. Establishing accurate reference values for leptin, especially in growing children and adolescents, is essential for the effective interpretation of its levels in physiological and various pathological states. These reference values differ significantly between adults and children due to differences in body composition, growth patterns, and metabolic demands [7,8,9].

Leptin exerts its physiological effects by binding to its receptor (OB-R), which is expressed in several tissues. OB-R belongs to class I cytokine receptors and exists in five forms (OB-Ra, OB-Rb, OB-Rc, OB-Rd, OB-Re) [10]. While OB-Ra–OB-Rd are membrane receptors, OB-Re is a soluble leptin receptor (sOB-R), not associated with cell membranes, reported to be the main leptin binding protein. The free leptin index (FLI), calculated as the ratio of leptin to sOB-R concentrations, has been proposed as a marker of free leptin in the circulation [11]. It reflects the balance between free and bound leptin, providing insights into leptin sensitivity and resistance. Elevated leptin levels observed in obesity are often associated with a simultaneous decrease in sOB-R levels, resulting in increased FLI values. Consequently, FLI may serve as a valuable indicator of metabolic health, offering potential implications for diagnosing and managing conditions such as obesity, diabetes, and other metabolic disorders [12].

In clinical practice, deviations from the established leptin reference ranges can indicate the presence of metabolic disorders such as obesity, lipodystrophy, and eating disorders. Elevated leptin levels are commonly associated with obesity and metabolic syndrome, where leptin resistance impairs its regulatory functions, contributing to further metabolic derangements [13]. Conversely, low leptin levels can be observed in conditions such as anorexia nervosa, malnutrition, and certain forms of lipodystrophy, highlighting the hormone’s role in energy deficiency states [ 14, 15, 16]. Moreover, leptin and its receptor have been implicated in various other diseases beyond metabolic disorders. Altered leptin signaling is observed in type 2 diabetes, contributing to insulin resistance and β-cell dysfunction [17]. Cardiovascular diseases have also been linked to leptin, with evidence suggesting its involvement in atherogenesis and hypertension [18].

In pediatric populations, leptin’s role extends to growth and development, with aberrant levels potentially contributing to conditions such as growth hormone deficiencies, delayed puberty, and developmental delays [19,20]. A potential predictive role of leptin or FLI has also been suggested in the diagnosis and monitoring of therapy for various diseases in children and adolescents [21,22,23]. Understanding the nuances of leptin physiology in children can be crucial for diagnosing and managing pediatric endocrine and metabolic disorders.

The aim of this study was to assess the values of leptin, leptin receptor, and FLI depending on age and sex in healthy Polish children, taking into account BMI, BMI Z-score, and fat mass.

## 2. Materials and methods

### 2.1. Patients

A total of 236 healthy children and adolescents aged 1–18 years were recruited to the study group between December 2019 and April 2023. Physical examination, including body height and weight measurements, were performed and BMI was calculated. Densitometric examination was performed in the group of 114 children aged 5 to 10 years. On the basis of data from the history and medical examination, these children were: (a) without either acute or chronic disorders, among them obesity; (b) not taking any medications that could affect their growth, pubertal development, nutritional or dietary status; (c) whose parents signed the informed consent form. All of the participants were Caucasian. Pubertal stage was determined according to the Tanner scale by the physician during the medical visit.

Physical examinations, including body height and weight measurements, were performed in all children and adolescents. Body Mass Index (BMI) was calculated as body weight divided by height squared (kg/m^2^). The BMI of each individual was converted to a standard BMI Z-score for the child’s age and sex using Polish reference tables [24]. Body composition was measured in 114 children aged 5 to 10 years by dual-energy X-ray absorptiometry (DXA) using Lunar Prodigy (General Electric Healthcare, Madison, WI, USA) with pediatric software version 9.30.044. All subjects were measured on the same machine. The measurements were performed using standard positioning techniques. The study was conducted in accordance with the Helsinki Declaration for Human Research, and the study protocol was approved (protocol code no. 9/18) by the Ethics Committee of the Institute of Mother and Child in Warsaw, Poland. Written informed consent was obtained from the parents of all the examined children.

### 2.2. Biochemical methods

Venous blood samples were collected between 8:00 and 10:00 a.m. after an overnight fast, and centrifuged at 1000 x g for 10 min at 4°C. Serum specimens were stored at −70°C prior to assay (no longer than six months). Biochemical parameters were determined by immunoenzymatic methods (ELISA). Commercially available ELISA kits (DRG Diagnostics, Marburg, Germany) were used to determine leptin and leptin receptor concentrations. The intra- and inter-assay CVs were less than 7.3% and 9.1% for leptin, and 7.2% and 9.8% for sOB-R, respectively. Analysis of each parameter was performed in duplicate. The FLI value was calculated as leptin concentration divided by sOB-R concentration.

### 2.3. Statistical analyses

Statistical analysis was performed using Statistica 6.0 (StatSoft Inc.) software. The Kolmogorov-Smirnov test and graphical inspections of data were used to evaluate distribution for normality. The results are presented as medians and interquartile ranges (25^th^–75^th^ percentiles) for non-normally distributed variables. Differences in the anthropometric characteristics and adipokine values of healthy girls and boys were assessed using the non-parametric Mann-Whitney *U* test. Spearman correlations between the anthropometric and studied adipokines were calculated. Differences were regarded as statistically significant at p<0.05.

## 3. Results

Data on the studied adipokines in 236 subjects (114 girls and 122 boys) are shown in Table 1. The groups of girls and boys were of similar age and did not differ in terms of weight, height, BMI, BMI Z-score values, or leptin receptor concentrations. Leptin concentrations and FLI were higher by about 50% (p=0.006; p=0.051, respectively) in girls than boys. Positive correlations were found in the whole study group between leptin and age, BMI, and BMI Z-score values (r=0.562, r=0.563, p=0.397, p<0.001, respectively) and even stronger between FLI and age, BMI, and BMI Z-score values (r=0.670, r=0.632, p=0.409; p<0.001, respectively). Negative correlations were found in the whole study group between leptin receptor and age, BMI, and BMI Z-score values (r=−0.711, r=−0.545, p=−0.252; p<0.001, respectively). Characteristics of the study population with age and sex stratification are presented in Table 2. Results are presented as medians, and interquartile ranges (25^th^–75^th^ percentiles) of biochemical parameters. With age, we observed a tendency towards higher leptin and FLI values in girls compared to boys. The observed leptin values varied significantly in adolescents aged 16–18 years old with sex differences (p=0.004). For FLI we found borderline significance (p=0.053) for this age group. We also analyzed the studied adipokines in three age groups: 1–4 years, 5–10 years, and in the group aged 11–18 years. The results obtained in children aged 1–4 years (32 girls, 37 boys) did not show differences in anthropometric parameters and adipokine values between girls and boys. Only in girls we found significant positive correlations between leptin and age (r=0.481, p=0.010) as well as FLI and age (r=0.437, p=0.015). Data concerning the group of prepubertal children aged 5 to 10 years (54 girls, 60 boys) are presented in Table 3. Age, BMI, BMI Z-score, and the analyzed adipokines did not show any differences between the two sex groups. However, we found higher values of fat mass (kg) and percentage of fat mass in girls than in boys (p=0.004, p=0.025, respectively). In the whole group of prepubertal children, we observed significant positive correlations between leptin and BMI, BMI Z-score, fat mass (kg), and percentage of fat mass (r=0.590, p<0.001; r=0.518, p<0.001; r=0.586, p<0.001; r=0.490. p<0.001, respectively), and negative correlations between leptin receptor and BMI, BMI Z-score, fat mass (kg), and percentage of fat mass (r=−0.382, p<0.001; r=−0.262, p=0.005; r=−0.549, p<0.001; r=−0.426. p=0.001, respectively). Positive associations were observed between FLI and BMI, BMI Z-score, fat mass in kilograms, and percentage of fat mass (r=0.600, p<0.001; r=0.496, p<0.001; r=0.648, p<0.001; r=0.538, p<0.001).

**Table 1.** Leptin, sOB-R, and FLI in the studied populations of children and adolescents.

**Variable**	**Girls n=114**	**Boys n=122**	**Total n=236**	***p*-value**
Age (years)	7.7 (5.2–10.1)	6.5 (5.0–9.1)	7.0 (5.1–9.8)	0.118
Weight (kg)	24.0 (17.7–33.2)	21.6 (17.4–32.6)	22.9 (17.6–33.1)	0.287
Height (cm)	125.9 (111.9–142.8)	122.0 (109.9–137.0)	122.5 (110.1–139.0)	0.270
BMI (kg/m^2^)	15.6 (14.5–17.3)	15.4 (14.4–16.9)	15.5 (14.4–17.2)	0.602
BMI Z-score	−0.43 (−0.89–0.19)	−0.49 (−0.94–0.00)	−0.46 (−0.91–0.12)	0.273
Leptin (ng/mL)	3.60 (1.60–6.35)	2.40 (1.43–3.90)	2.75 (1.60–5.15)	0.006
sOB-R (ng/mL)	39.3 (30.5–51.6)	40.0 (29.4–52.4)	39.7 (30.0–52.3)	0.982
FLI	0.077 (0.03–0.20)	0.052 (0.03–0.14)	0.057 (0.03–0.15)	0.051

Data are presented as medians and interquartile ranges (25^th^–75^th^); FLI – leptin/leptin receptor ratio; sOB-R – soluble leptin receptor; BMI – body mass index.

**Table 2.** Leptin, sOB-R, and FLI in children and adolescents aged 1–18 years old stratified by age, sex, and BMI.

**Parameter/years**	**Girls (n=114)**					**Boys (n=122)**				

	**n (Girls/Boys)**	**BMI (kg/m^2^)**	**Percentiles**			**BMI (kg/m^2^)**	**Percentiles**			***P*-value**
	
			**25**	**50**	**75**		**25**	**50**	**75**	
Leptin (ng/mL)										
1–3	12/18	15.2 (13.8–15.7)	0.79	1.12	1.30	14.7 (14.2–16.6)	1.01	1.31	1.83	0.280
4–6	39/40	14.7 (13.8–15.9)	1.49	2.10	3.19	14.8 (13.9–15.4)	1.57	2.04	2.90	0.861
7–9	32/33	15.7 (14.7–17.2)	2.77	4.90	7.03	15.9 (14.7–17.2)	1.60	3.10	5.83	0.104
10–12	11/10	16.7 (15.2–17.5)	3.80	4.40	7.60	16.3 (16.1–18.5)	1.09	3.05	5.00	0.107
13–15	10/10	19.6 (17.5–21.5)	6.30	7.10	10.87	18.5 (18.0–21.4)	3.05	4.00	7.30	0.055
16–18	10/11	20.3 (19.0–22.1)	7.95	10.24	14.00	20.9 (20.3–20.9)	2.80	3.20	5.10	0.004
sOB-R (ng/mL)										
1–3	12/18	15.2 (13.8–15.7)	48.9	55.9	61.5	14.7 (14.2–16.6)	50.3	61.0	69.7	0.374
4–6	39/40	14.7 (13.8–15.9)	41.0	48.6	65.2	14.8 (13.9–15.4)	37.9	48.1	57.4	0.330
7–9	32/33	15.7 (14.7–17.2)	31.0	38.1	44.4	15.9 (14.7–17.2)	26.8	32.7	41.7	0.142
10–12	11/10	16.7 (15.2–17.5)	29.0	32.0	34.1	16.3 (16.1–18.5)	26.8	28.4	35.2	0.161
13–15	10/10	19.6 (17.5–21.5)	17.2	24.5	26.8	18.5 (18.0–21.4)	21.1	22.9	26.3	0.848
16–18	10/11	20.3 (19.0–22.1)	19.5	20.8	24.5	20.9 (20.3–20.9)	17.2	17.4	19.8	0.072
FLI										
1–3	12/18	15.2 (13.8–15.7)	0.01	0.02	0.03	14.7 (14.2–16.6)	0.02	0.02	0.03	0.626
4–6	39/40	14.7 (13.8–15.9)	0.02	0.05	0.06	14.8 (13.9–15.4)	0.03	0.04	0.06	0.936
7–9	32/33	15.7 (14.7–17.2)	0.05	0.14	0.20	15.9 (14.7–17.2)	0.04	0.08	0.16	0.189
10–12	11/10	16.7 (15.2–17.5)	0.08	0.14	0.21	16.3 (16.1–18.5)	0.04	0.11	0.15	0.297
13–15	10/10	19.6 (17.5–21.5)	0.24	0.40	0.62	18.5 (18.0–21.4)	0.12	0.18	0.30	0.085
16–18	10/11	20.3 (19.0–22.1)	0.28	0.54	9.77	20.9 (20.3–20.9)	0.14	0.16	0.33	0.053

Data are presented as medians and interquartile ranges (25^th^–75^th^); sOB-R – soluble leptin receptor; FLI – leptin/leptin receptor ratio; BMI – body mass index.

**Table 3.** Leptin, sOB-R, FLI, and anthropometric parameters in prepubertal healthy children aged 5–10 years old

**Variable**	**Girls n=54**	**Boys n=60**	**Total n=114**	***p*-value**
Age (years)	7.9 (6.3–9.1)	7.0 (5.5–9.0)	7.5 (5.7–9.0)	0.109
Weight (kg)	23.6 (18.5–28.8)	22.0 (18.2–27.7)	23.1 (18.3–28.5)	0.731
Height (cm)	124.3 (115.3–133.3)	122.5 (113.0–132.0)	123.0 (114.1–132.9)	0.494
BMI (kg/m^2^)	15.3 (14.4–16.4)	15.4 (14.6–16.4)	15.3 (14.5–16.4)	0.527
BMI Z-score	−0.44 (−0.75–0.11)	−0.32 (−0.78–0.08)	−0.38 (−0.77–0.11)	0.748
Leptin (ng/mL)	3.65 (1.60–5.93)	2.75 (1.60–4.75)	3.00 (1.60–5.33)	0.212
sOB-R (ng/mL)	40.2 (33.0–52.4)	40.7 (32.6–51.0)	40.5 (32.7–51.5)	0.714
FLI	0.08 (0.04–0.15)	0.05 (0.03–0.13)	0.06 (0.03–0.14)	0.317
Fat mass (kg)	4.87 (3.33–6.71)	3.63 (2.78–5.38)	4.23 (2.96–6.54)	0.004
Fat mass (%)	23.1 (19.0–27.1)	18.6 (14.2–23.0)	21.6 (16.2–25.4)	0.025

Data are presented as medians and interquartile ranges (25^th^–75^th^); sOB-R – soluble leptin receptor; FLI – leptin/leptin receptor ratio; BMI – body mass index.

In the girls and boy groups separately, we obtained positive correlations between leptin and BMI (r=0.589, p<0.001, r=0.615, p<0.001, respectively), BMI Z-score (r=0.520, p<0.001; r=0.529, p<0.001, respectively), fat mass (kg) (r=0.580, p<0.001; r=0.570, p<0.001, respectively), and percentage of fat mass (r=0.443, p<0.001; r=0.475, p<0.001, respectively). Negative correlations were also found in the girl and boy groups between leptin receptor and BMI (r=−0.364, p=0.007; r=−0.372, p=0.004, respectively), BMI Z-score (only for boys r=−0.284, p=0.028), fat mass (kg) (r=−0.497, p<0.001; r=−0.651, p<0.001, respectively), and percentage of fat (r=−0.299, p=0.030; r=−0.560, p<0.001, respectively).

**Figure 1.**
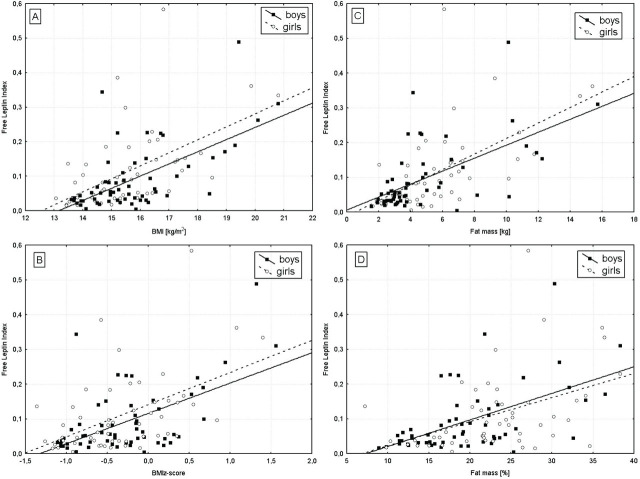
Correlations of FLI with BMI (A), BMI Z-score (B), fat mass (kg) (C), and fat mass (%) (D) in girls and boys aged 5–10 years old.

In the groups of prepubertal girls and boys, we found positive correlations between FLI and BMI (r=0.692, p<0.001; r=0.571, p<0.001, respectively) (Fig. 1A), BMI Z-score (r=0.639, p<0.001; r=0.530, p<0.001, respectively) (Fig.1B), fat mass (kg) (r=0.632, p<0.001; r=0.465, p<0.001, respectively) (Fig.1C), and percentage of fat mass (r=0.622, p<0.001; r=0.687, p<0.001, respectively) (Fig.1D).

Data concerning the group of adolescents (28 girls, 25 boys) are presented in Table 4. Age, BMI, BMI Z-score, and leptin receptor concentrations did not show any differences between the two sex groups. However, higher leptin concentrations (p=0.001) and FLI values (p=0.005) were observed in girls than in boys. In the whole group of adolescents, we observed significant positive correlations between leptin and age (r=0.362, p=0.008), and leptin and BMI (r=0.327, p=0.017). Negative correlations between leptin receptor and age, BMI, and BMI Z-score (r=−0.582, p<0.001; r=−0.520, p<0.001; r=−0.287, p=0.037, respectively) were found. Additionally, positive associations were observed between FLI and age, BMI, and BMI Z-score (r=0.443, p<0.001; r=0.449, p<0.001; r=0.310, p=0.024, respectively).

**Table 4.** Leptin, sOB-R, FLI, and anthropometric parameters in healthy adolescents aged 11–18 years old

**Variable**	**Girls n=28**	**Boys n=25**	**Total n=53**	***p*-value**
Age (years)	13.8 (12.0–17.0)	15.0 (12.7–16.0)	13.9 (12.0–16.0)	0.694
Weight (kg)	48.0 (37.0–59.2)	57.0 (41.3–65.0)	50.5 (40.3–61.8)	0.076
Height (cm)	162.0 (151.0–166.0)	174.0 (161.0–179.0)	164.5 (153.5–172.0)	0.026
BMI (kg/m^2^)	18.4 (17.4–20.3)	18.9 (18.1–20.9)	18.6 (17.6–20.7)	0.634
BMI Z-score	−0.28 (−0.74–0.12)	0.03 (−0.70–0.29)	−0.21 (−0.73–0.20)	0.822
Leptin (ng/mL)	7.10 (4.40–11.30)	3.90 (2.80–5.40)	5.40 (3.83–9.10)	0.001
sOB-R (ng/mL)	25.1 (19.5–30.0)	22.3 (19.6–26.8)	24.8 (19.5–28.6)	0.141
FLI	0.28 (0.16–0.54)	0.16 (0.13–0.20)	0.21 (0.14–0.40)	0.005

Data are presented as medians and interquartile ranges (25^th^–75^th^); sOB-R – soluble leptin receptor; FLI – leptin/leptin receptor ratio; BMI – body mass index.

Analyzing the girls and boy groups separately, we obtained positive correlations between leptin and age as well as BMI (r=0.479, p=0.010; r=0.459, p=0.014, respectively) only for girls. Also, in girls we found positive correlations between FLI and age (r=0.516, p=0.005), BMI (r=0.601, p=0.001), and BMI Z-score (r=0.415, p=0.028). Negative correlations were found in girls and boys between leptin receptor and age (r=−0.568, p=0.002; r=−0.727, p<0.001, respectively), BMI and BMI Z-score (only in girls r=−0.520, p<0.00; r=−0.287, p=0.037, respectively).

## 4. Discussion

This study presents serum values of leptin, leptin receptor, and FLI in healthy Polish children and adolescents aged 1–18 years, showing the relations between the studied parameters and age, sex, and BMI in this population. We observed higher concentrations of leptin and FLI values in girls than in boys, similar to other authors [9,25,26,27,28,29]. Additionally, we obtained an increase of leptin concentrations and FLI with age and BMI. The values of these parameters had a tendency to be higher in girls than in boys. This also may apply to the prepubertal period, when leptin and FLI values in girls were slightly higher than in boys. In boys, the highest values of leptin and FLI were found at 13–15 years and then lowered. Other authors observed an increase of leptin concentrations with age in girls, but a bell-shaped association with age in boys [25,26]. It is known that estradiol may have a stimulating effect on leptin levels in men, whereas testosterone has a suppressive effect [30]. Therefore, the decreased leptin values observed in postpubertal males may be explained by the rising testosterone levels during puberty, which then leads to a decrease in serum leptin levels [25].

Similar to previous studies, we also found positive associations between leptin and age, BMI, BMI Z-score, and fat mass in all children aged 5 to 10 years and in girls and boys separately [25,26,30,31]. The values of fat mass are higher in prepubertal girls than in boys. In addition, slightly higher leptin levels in girls may be associated with differences in body composition between girls and boys observed during the prepubertal period.

The weight-regulating effects of leptin are mediated by binding and activation of the specific receptor in the brain, while sOB-R modulates leptin levels in the circulation and prevents this hormone from degradation [32]. In our study, we did not observe any differences in the sOB-R receptor concentration depending on sex, but we found a gradual decrease in the receptor value with age in both girls and boys. Negative associations between sOB-R and age as well as anthropometric parameters in healthy children and adolescents were observed by us and other authors [26].

FLI values in the prepubertal group and in the subgroups of girls and boys showed a positive correlation with BMI, BMI-Z, and fat mass. It is worth highlighting that these correlations were even stronger than the correlations between anthropometric parameters and leptin as well as sOB-R separately. Moreover, the relations between FLI and BMI, BMI Z-score, and fat mass were also stronger in girls than in boys in the prepubertal group, probably due to the observed sex-related differences in body composition.

It is known that leptin or leptin receptor levels even in healthy children can be influenced by genetic and/or metabolic factors. The diverse response of leptin levels and body mass changes depending on polymorphic variants of LEP and LEPR has been investigated [33,34,35]. There are studies suggesting a gene-gene interaction between the LEP and LEPR variants in a genetic susceptibility to the development of obesity [36]. Besides them, some authors observed alternations of serum leptin concentration due to overnutrition or undernutrition in the perinatal period, which may determine metabolic changes during childhood as well as adulthood [31]. The higher leptin concentrations in non-obese healthy subjects might be the result of nutritional programming through maternal undernutrition or obesity and even periconceptional maternal obesity [37,38,39]. Hence, high concentrations of sOB-R in early childhood may at least in part suppress leptin actions on its membrane receptors [26]. It is suggested that leptin and leptin receptor as FLI may help to evaluate phenotypically non-obese children by detecting those with more unfavorable risk profiles, independent of BMI values [40,41]. This index corresponds with leptin action, determines leptin resistance, and is known to be a better indicator of metabolic complications than leptin levels alone [21,42,]. According to Catli et al. [21] FLI may be a marker to evaluate leptin resistance in obese children and increased FLI may contribute toward the development of hyperinsulinemia and insulin resistance. It is also described that free leptin may play a role as predictive factor of cardiac function in pediatric patients with chronic kidney disease [23]. Moreover, FLI may identify the changes in metabolism associated with weight loss in obese children during lifestyle intervention programs [34,43,44,45]. Longitudinal observations of the studied patients may confirm the usefulness of this index in the management of obese children and adolescents.

It is a strength of this study that the values in healthy children and adolescents covered the almost full pediatric age span (1–18 years). Additionally, the blood samples were drawn in the morning following an overnight fast, minimizing the circadian influence of adipokines on the samples [46]. Another strength was the evaluation of not only leptin and leptin receptor concentrations separately but also FLI values, which corresponds with the action of free leptin crossing the blood-brain barrier and affecting appetite and energy expenditure. Our results present the relations between adipokines and age, sex, BMI, and additionally fat mass in prepubertal children. A limitation of this study is the lack of measurement of fat mass in the whole study group and the relatively small number of healthy children and adolescents. However, none of the approaches could diminish the associations of age and BMI on adipokine levels.

In conclusion, we present the results concerning age-, sex-, and BMI- dependent leptin and leptin receptor concentrations and FLI values in healthy Polish children and adolescents. The presented results may be useful for the standardization of adipokine values in order to compare these parameters between healthy children and adolescents with various disorders, such as obesity and obesity-related diseases. These data may also be used in prevention for the early identification of children with an unfavorable adipokine profile resulting in predisposition to the development of obesity and obesity-related complications. In clinical practice, these markers may be helpful in monitoring therapy effectiveness in patients with metabolic diseases, among them obesity, diabetes, and metabolic syndrome.
